# Self-reported poor oral hygiene among in-school adolescents in Zambia

**DOI:** 10.1186/1756-0500-4-255

**Published:** 2011-07-22

**Authors:** Seter Siziya, Adamson S Muula, Emmanuel Rudatsikira

**Affiliations:** 1 Department of Community Medicine, University of Zambia, Medical School, Lusaka, Zambia; 2 Department of Community Health, University of Malawi, College of Medicine, Blantyre, Malawi; 3 School of Community and Environmental Health, Old Dominion University, Norfolk, Virginia, USA

## Abstract

**Background:**

Dental health is a neglected aspect of adolescent health globally but more so in low-income countries. Secondary analysis using the 2004 Zambia Global School-Based Health Survey (GSHS) was conducted in which we estimated frequencies of relevant socio-demographic variables and explored associations between selected explanatory variables and self-reported poor oral hygiene (not cleaning or brushing teeth) within the last 30 days of the completion of questionnaire.

**Findings:**

Most of the 2257 respondents were males (53.9%) and went hungry (82.5%). More than 4 in 10 respondents drank alcohol (42.2%) while 37.2% smoked cannabis. Overall 10.0% of the respondents reported to have poor oral hygiene. Male respondents were 7% less likely to report to have poor oral hygiene compared to females. Compared to respondents who never drank alcohol, those who drank alcohol were 27% more likely to report to have poor oral hygiene. Respondents who smoked cannabis were 4% more likely to report to have poor oral hygiene compared to those who did not smoke cannabis. Finally, respondents who went hungry were 35% more likely to report to have poor oral hygiene compared to those who did not go hungry.

**Conclusions:**

Results from this study indicate that female gender, alcohol drinking, cannabis smoking, and going hungry were associated with self-reported poor oral hygiene. The identification of these factors should guide the design and implementation of programs aimed to improve oral health among adolescents.

## Background

Dental health has received limited attention in many countries in sub-Saharan Africa. Despite the paucity of data of dental health in most African countries, some reports are worth highlighting. Folayan et al have reported that DMFT (decayed, missing and filled teeth) was negatively associated with age but positively associated with sugar intake in a sample of Nigerian children [1]. In a study of South African children, 52% of the children with early childhood caries (ECC) were reported not to have been supervised during tooth brushing [2]. Masanja and Mumghamba have reported that, in Tanzania, secondary school adolescents aged 13 to 17 years had partial knowledge about gingivitis but a good knowledge of the basic oral hygiene measures necessary to maintain proper oral health [3]. About 99% of the study participants perceived that it was necessary to brush their teeth on a daily basis.

There are limited published reports on oral health in Zambia. Noar and Portnoy have suggested that the changing social and economic situation for most families, resulting in high sugar intake in Zambia were influencing the epidemiology of dental conditions in the country [4]. Meanwhile, Dickson highlighted the reduced prevalence of dental caries in western province of Zambia partly due to almost no availability of refined sugar and use of wood ash to clean teeth [5]. Baboo et al have corroborated this finding and reported that urbanization and confectionary eating were contributing to the increased dental pathology among children in Zambia [6]. Tooth brushing is the primary recommended practice for maintaining dental hygiene [7]. It is, therefore, important that persons with poor oral hygiene are identified and interventions instituted to improve their oral health.

We used data from the Zambia Global School-Based Health Survey (GSHS) conducted in 2004 to estimate the prevalence of self-reported poor oral hygiene and assess the socio-demographic factors that were associated with the practice.

## Methods

Details of the Zambia GSHS of 2004, especially with regard to the sampling strategy, have been published elsewhere [8,9]. A two-stage sampling technique was used to recruit students mainly aged 13 to 15 years in schools. In the first stage of sampling, schools with grades having 13-15 years olds were included in a sampling frame. Each school's chance of being selected was proportional to its enrollment size. In the second stage of sampling, a systematic sample of classes within each school was obtained. The survey achieved a response rate of 75%.

Both the Ministries of Health and Education provided ethical oversight of the Zambia GSHS. Although eligible students were encouraged to participate, they were also informed that they were free not to participate or decide not to answer any of the questions they felt uncomfortable with. Questionnaires, designed in a multiple choice fashion, were self-completed in class by the students within one school period i.e. about 40-50 minutes. The questionnaire has been used in over 50 countries, and as such it has been validated. No follow-up sessions were conducted for students who were absent from school on the day the questionnaire was administered.

The main question was: "During the past 30 days, how many times per day did you usually clean or brush your teeth?" Students were given the option of replying: I did not clean or brush my teeth during the past 30 days; 1 time per day; 2 times per day; 3 times per day; 4 or more times per day. All respondents who reported "I did not clean or brush my teeth during the past 30 days" were coded "1" i.e. did not brush teeth, while those who reported at least 1 time were coded as "0" i.e. positive history of reporting ever brushed teeth in the past 30 days. The category of no cleaning or brushing teeth was termed as self-reported poor oral hygiene. No clinical parameters or indices of plague or gingival status were collected.

The Statistical Package for the Social Sciences (SPSS) version 11.5 (Chicago Illinois, United States was used for the analysis. We obtained frequencies and proportions of the outcome variable (self-reported poor oral hygiene) and some key variables. The Pearson's Chi-square test was used to determine associations between the explanatory variables and the outcome variable during bivariate analyses. The cut off point for statistical significance was set at the 5% level. A Backward logistic regression analysis was conducted to estimate the magnitudes of associations (Adjusted Odds Ratios [AOR] with 95% Confidence Intervals [CI]) between the outcome variable and the following explanatory variables that were identified in the literature to be significantly associated with poor oral hygiene and collected in the survey: sex, age, drank alcohol, smoked cannabis, and went hungry. The questions were: During the past 30 days, on how many days did you have at least one drink containing alcohol?; During your life, how many times have you used drugs cannabis?; During the past 30 days, how often did you go hungry because there was not enough food in your home? The last question was used as an indirect measure of poverty or household socio-economic status. No other socio-economic variables such as parental income, number of siblings and housing status were collected.

### Description of the sample for Zambian sample

A total of 2257 respondents took part in the study. Most of the respondents were male (53.9%), drank alcohol (42.2%), ever smoked cannabis (37.2%), and went hungry (82.5%). Most of the male respondents were aged 16 years or more (36.5%), whilst most female respondents were 13 years olds or younger (30.9%). Overall 10.0% of the respondents reported to have poor oral hygiene. The description of the sample is shown in Table 1.

**Table 1:** Description of the sample for the 2004 Zambian Global School Health Survey

	Total	Male	Female
Factor	n* (%)**	n* (%)**	n* (%)**
Age			
< 14	463 (27.5)	156 (21.5)	263 (30.9)
14	386 (19.1)	156 (17.4)	219 (21.6)
15	513 (22.8)	260 (24.5)	238 (21.6)
16+	708 (30.6)	394 (36.5)	306 (25.9)
Sex			
Male	994 (53.9)	-	-
Female	1039 (46.1)	-	-
Drank alcohol			
Yes	528 (42.2)	217 (38.5)	283 (45.1)
No	805 (57.8)	388 (61.50	388 (54.9)
Smoked cannabis			
Yes	695 (37.2)	300 (34.5)	357 (39.4)
No	1301 (62.8)	634 (65.5)	614 (60.6)
Went hungry			
Yes	1691 (82.5)	787 (82.3)	834 (83.4)
No	366 (17.5)	173 (17.7)	170 (16.6)
Self reported poor oral hygiene			
Yes	186 (10.0)	81 (10.3)	96 (9.8)
No	1891 (90.0)	884 (89.7)	920 (90.2)

* unweighted frequency

** weighted percent

Numbers may not add up due to missing information.

### Correlates for self-reported poor oral hygiene among Zambian school-going adolescents

Table 2 shows results from bivariate analyses. All the factors (age, sex, drank alcohol, smoked cannabis and went hungry) were significantly associated with self-reported poor oral hygiene. Respondents reporting poor oral hygiene tended to be younger than 14 years, males, drank alcohol, smoked cannabis and went hungry.

**Table 2:** Factors associated with self-reported poor oral hygiene in bivariate analyses

	Self-reported poor oral hygiene		
		
Factor	No* (%)**	Yes* (%)**	p value
Age			
< 14	58 (37.2)	392 (26.5)	< 0.001
14	34 (18.8)	341 (19.2)	
15	30 (13.8)	470 (23.9)	
16+	61 (30.2)	627 (30.4)	
Sex			
Male	81 (54.8)	884 (53.6)	< 0.001
Female	96 (45.2)	920 (46.4)	
Drank Alcohol			
Yes	55 (59.0)	459 (40.7)	< 0.001
No	48 (41.0)	739 (59.3)	
Smoked cannabis			
Yes	82 (49.4)	590 (35.5)	< 0.001
No	93 (50.6)	1179 (64.5)	
Went hungry			
Yes	154 (87.6)	1485 (81.7)	< 0.001
No	23 (12.4)	339 (18.3)	

* unweighted frequency

** weighted percent

Numbers may not add up due to missing information.

The results of multivariate analysis are shown in Table 3. Although age remained significantly associated with self-reported poor oral hygiene during the multivariate analysis, it was not consistently associated with the outcome. While respondents who were aged less than 14 years (AOR = 0.74, 95%CI [0.72, 0.77]) and those of age 15 years (AOR = 0.74, 95%CI [0.71, 0.76]) were less likely to report to have poor oral hygiene, those of age 14 years (AOR = 1.51, 95%CI [1.47, 1.56]) were more likely to report to have poor oral hygiene compared to respondents aged 16 years or older. Male respondents were 7% (AOR = 0.93, 95%CI [0.91, 0.94]) less likely to report to have poor oral hygiene compared to female respondents. Compared to respondents who never drank alcohol, those who drank alcohol were 27% (AOR = 1.27, 95%CI [1.25, 1.30]) more likely to report to have poor oral hygiene. Respondents who smoked cannabis were 4% (AOR = 1.04, 95%CI [1.02, 1.07]) more likely to report to have poor oral hygiene. Finally, adolescents who went hungry were 35% (AOR = 1.35, 95%CI [1.31, 1.39]) more likely to report to have poor oral hygiene compared to the respondents who did not go hungry. These results are shown in Table 3.

**Table 3:** Correlates for self reported poor oral hygiene among Zambian school-going adolescents

Factor	AOR* (95%CI**)
Age	
< 14	0.74 (0.72, 0.77)
14	1.51 (1.47, 1.56)
15	0.74 (0.71, 0.76)
16+	1
Sex	
Male	0.93 (0.91, 0.94)
Female	1
Drank alcohol	
Yes	1.27 (1.25, 1.30)
No	1
Smoked cannabis	
Yes	1.04 (1.02, 1.07)
No	1
Went hungry	
Yes	1.35 (1.31, 1.39)
No	1

AOR* Adjusted Odds Ratio

CI** Confidence Interval.

## Discussion

In a survey of in-school adolescents in Zambia in 2004, 10.0% reported to have poor oral hygiene in the 30 days preceding the survey. In multivariable logistic regression analysis, age, sex, substance use (alcohol, cannabis) and going hungry were significantly associated with self-reported poor oral hygiene.

Although age was significantly associated with self-reported poor oral hygiene, the results from this study do not suggest a specific pattern as only the 14 years olds group had higher odds of not brushing while the other age groups had lower odds compared to 16 years of age or older. We offer no explanation for this finding.

Although, girls reported significantly more frequent optimal oral hygiene practice than boys in Australia [10] and Kenya [11], on the contrary, female respondents in our study were more likely to report to have poor oral hygiene. However, unhealthy lifestyles such as substance use was associated with self-reported poor dental hygiene, highlighting that multiple unhealthy practices may converge within the same individual [9]. Finally, if we assumed that having gone hungry because of lack of food in the household is some measure of poverty, it would suggest, from our results that poverty was positively associated with self-reported poor oral hygiene. This finding i.e. that adolescents from poor households are less likely to attend to their dental hygiene or have poor dental health has also been reported by other authors [12,13].

This study has a number of limitations. Being a cross sectional design, we may not be able to assign causation to any of the factors. To the extent that survey participants mis-reported, our results may be biased. We have no specific information to suggest that survey participants may have mis-reported or not on any of the variables. There were some missing data, and these were declared missing in the analysis. The relatively low response rate may have biased our results. However, we do not have data on the non-respondents that we may use to compute the magnitude and direction of the bias. Furthermore, only adolescents in school were eligible for recruitment. The findings may therefore be applicable to adolescents who are in school but not drop-outs and those who have never been in school. Finally, no clinical parameters or indices of plaque or gingival status were collected.

## Conclusion

Results from this study indicate that female gender, alcohol drinking, cannabis smoking, and going hungry were positively associated with self-reported poor oral hygiene. The identification of these factors should guide the design and implementation of school and community-based programs aimed to improve oral health among adolescents. Some of the programs may include educational and awareness outreach through media and youth social networks.

## Competing interests

The authors declare that they have no competing interests.

## Authors' contributions

SS led data analysis and interpretation, ER contributed to the interpretation of the data and ASM led the drafting of the manuscript. All authors read and approved the final manuscript.
